# Optimizing Harvesting Efficiency: Development and Assessment of a Pneumatic Air Jet Excitation Nozzle for Delicate Biostructures in Food Processing

**DOI:** 10.3390/foods13101458

**Published:** 2024-05-08

**Authors:** Carlos I. Cardona, Héctor A. Tinoco, Luis Perdomo-Hurtado, Eduardo Duque-Dussán, Jan Banout

**Affiliations:** 1 Experimental and Computational Mechanics Laboratory, Universidad Autónoma de Manizales, Edificio Fundadores, Manizales 170001, Colombia; carlosi.cardona@autonoma.edu.co (C.I.C.); htinoco@autonoma.edu.co (H.A.T.); lperdomo@autonoma.edu.co (L.P.-H.); 2 Institute of Physics of Materials, Sciences Academy of the Czech Republic, 61600 Brno, Czech Republic; 3 Central European Institute of Technology, 61600 Brno, Czech Republic; 4 Department of Sustainable Technologies, Faculty of Tropical AgriSciences, Czech University of Life Sciences Prague, Kamýcká 129, 16500 Prague, Czech Republic; duque_dussan@ftz.czu.cz

**Keywords:** precision nozzle design, pneumatic air jet excitation, shooting frequencies, contactless vibrations generation, food processing, harvesting efficiency

## Abstract

This study presents a new pneumatic air jet excitation nozzle, specifically designed for food processing applications. The device, which uses compressed air equipment and a precision solenoid valve, controls air discharge through a parametric air jet nozzle. Tests showed that the device could achieve shooting frequencies in the 40–45 Hz range, with operational pressures between 5 and 7 bar. A sensor system was used to measure the force generated by the device at different frequencies and pressures. Using the Design of Experiments (DOE) methodology, we identified optimal cavity designs for 5 and 6 bar pressures. These designs outperformed others in generating uniform force and maintaining consistent vibration voltage behavior. This highlights the efficacy of our approach in enhancing device performance under different conditions. The device’s practical application in food processing was demonstrated, particularly in delicate tasks such as the selective harvesting of sensitive crops like coffee fruits. The precise vibrations generated by the device could potentially enhance harvesting efficiency while significantly reducing mechanical damage to plants. The results position the device as a compelling proof of concept, offering an alternative method for exciting biostructures in food processing. This device opens up new possibilities in agricultural and biological fields, providing a non-intrusive and practical approach to manipulating and interacting with delicate, contactless structures, with a specific focus on improving food processing efficiency and quality.

## 1. Introduction

Several methods of contactless mechanical excitation have been studied, where ultrasound wave propagation [1], air [2], magnetic [3], and laser pulses [4] acted as energy sources. These have been proposed to generate vibrations over diverse kinds of structures. All these techniques are widely used to diagnose structural integrity, detect failure, characterize dynamic properties, and predict behavior [5,6,7,8]. For instance, a laser ablation excitation mechanism was developed [5] to characterize a rotating disk on a hard drive. This type of excitation uses a high-power YAG pulse laser that generates an impulse force on the disk surface, and vibrations were measured using a Doppler effect laser vibrometer. In a similar approach to designing a resonator, a laser diode excites the specimen, generating lateral vibrations and leading to four resonance frequencies between 0.1 and 100 kHz [9]. In the domain of acoustic approaches, various studies in the literature have employed a confocal ultrasound transducer (MicroAcoustics Instruments, Broad-band Air-Coupled Transducer sBAT-5) to stimulate a hard-disk suspension system within the 1 to 50 kHz range [8]. Notably, this research demonstrated that comparable amplitudes could be achieved through both mechanical and acoustic excitation, even when dealing with different vibrational modes. These energy transfer methods leverage ultrasonic transducers as their foundation. Moreover, researchers actively explore novel wireless acoustic energy transfer avenues utilizing vibration or ultrasound waves [10]. The acoustic excitation method also finds noteworthy applications in aerospace component noise analysis [11] and nondestructive materials analysis techniques [12]. Gas impact methods for transferring mechanical energy remain underexplored [13]; however, thermal energy dissipation has been achieved through directed airflow in targeted personalized ventilation [14].

The nozzle is the key component controlling the performance of devices designed to target specific vibrational responses via air shooting [6]. Parameters such as cavity length, inlet diameter, and outlet diameter directly affect the vortex transport capacity from cavities with actuators [15]. Currently, there is no evidence of a device that maximizes impact force solely by utilizing the energy in stored air pressure [16,17]. Typically, these methods involve the use of actuators to transfer the air. Therefore, the design of the air outlet nozzle must carefully consider factors like pressure, distance, and profile incidence to optimize the air dispersion phenomenon [18]. Additionally, researchers have explored alternative methods to generate air vortex rings capable of colliding with distant objects and a customized ventilation system based on triggered vortex rings, enabling the efficient long-distance delivery of fresh air with minimal dissipation [15]; or some experiments studied vortex formation, breakup, and regrouping using water-immersed nozzles [19].

Air pulse excitation devices have been implemented for the experimental modal analysis of small structures such as cantilever beams [6]. The device was developed for the purpose of characterizing micro-electro-mechanical systems (MEMS) and was validated in tests on a cantilever beam in a frequency range of 0 to 8 kHz. Furthermore, this approach has also been successfully employed for characterizing hard disks where significant forces of up to 0.6 N and excitations up to 1 kHz have been achieved through pressurized air excitation. Forces up to 0.6 N and excitations up to 1 kHz have been attained through pressurized air excitation [13]. The air pulses were applied in a range from 0 to 600 kPa, determining the relationship between the pressure and maximum force produced by the air excitation. The intensity of the force is directly influenced by variations in pressure, as well as the nozzle profile which creates dispersion in the airflow and affects the energy transferred from the jet to the target structure [18]. An exciter concept based on air-jet pulses was developed as a solution to perform non-contact experimental modal analysis (EMA) on small composite specimens [20]. The tests were performed between 0 and 500 Hz using frequency nonlinear sweep excitation. The proposed device achieved controlled dynamic conditions, and the contribution of mechanical impedance on the specimens was avoided while the nozzles were depicted to be one of the key elements to induce vibrations [20].

A Computational Fluid Dynamics (CFD) analysis of the three optimal designs, which was conducted in a previous study [21], involved simulating the most optimal nozzles under ideal conditions to obtain the velocity contour plot distribution. The CFD model results were further complemented by an experimental study of the jet morphometry, where the turbulence was analyzed by studying the sensitivity of the velocity and geometrical variation in the jet [21]. To comprehensively characterize the flow-induced vibrations generated by the pneumatic air jets, a device that enables the characterization of the vibrational consequences of the air impact is crucial, for example, flow-induced continuous-vibration sensing by implementing control performance of a multilayer beam structures containing pivot controls by means of an electromagnetic field [22].

This paper outlines the design, manufacturing process, and experimental assessment of a pressurized air excitation nozzle. Through the integration of pneumatic equipment and accessories, a novel air pulse excitation device was developed specifically for inducing vibrations in biological structures. To achieve a feasible nozzle design, two stages were performed: (1) evaluation of the operational conditions (pressure and frequency) and (2) geometrical factors (cavity length and outlet diameter). In addition, the impact force generated by the device was measured under a parametric design scheme. Then, the air jet discharge was studied by implementing a force and vibration sensing system. Additionally, we proposed a strategy for measuring the flow-induced vibrations by the nozzle using piezoelectric transducers bonded to a brass platform to sense vibrations and characterize the device’s performance. It aims to provide an alternative for the excitation of fragile structures that are highly sensitive to small excitation forces. The potential real-world applications of the device extend to mechanized fruit harvesting and similar agricultural practices. By exploring its performance in agricultural environments, new possibilities emerge for enhancing harvest efficiency while preserving the integrity of both the trees and the harvested fruits. For example, in coffee harvesting, where delicate handling is crucial for maintaining bean quality, the device offers a promising solution. Its gentle vibrations facilitate fruit detachment without causing mechanical damage to the tree or compromising the structure of the coffee fruits. Similarly, in harvesting operations for other crops like apples or citrus fruits, the device could revolutionize the process by enabling efficient harvesting while minimizing the impact on tree health and fruit quality. These applications highlight the device’s potential to significantly contribute to sustainable agricultural practices, reducing labor intensity, minimizing product damage, and optimizing harvest yields.

## 2. Materials and Methods

### 2.1. Parametric Design of the Air Jet Nozzle

**Figure 1:**
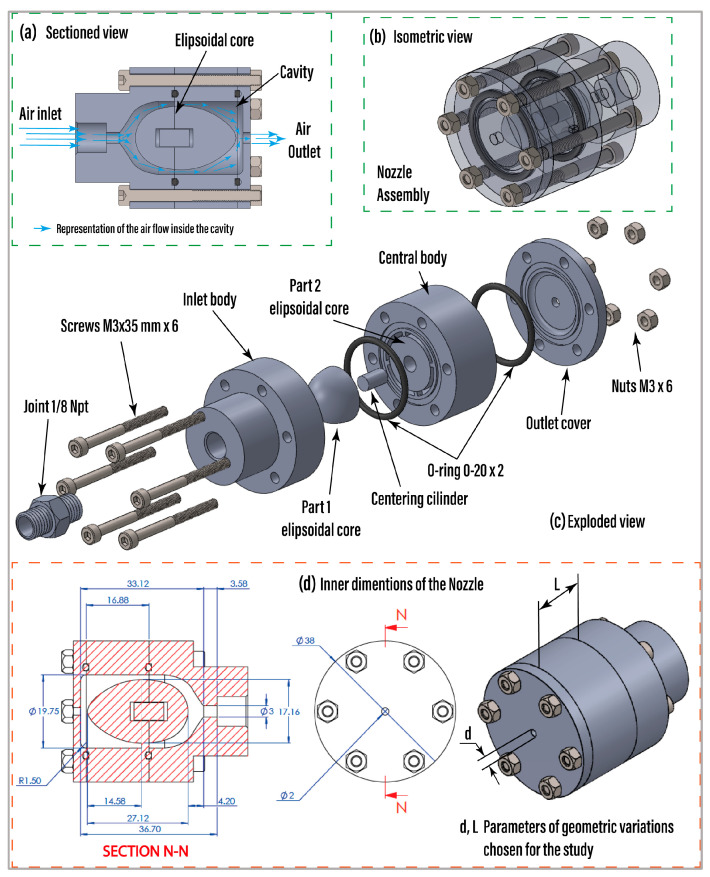
Nozzle operational design. (**a**) Cross-sectional view with the operational schematic. (**b**) Isometric view of the assembly. (**c**) Exploded view and parts. (**d**) Overall dimensions of the nozzle cavity and geometric parameters chosen for this study.

Prior to construction, the nozzles investigated in this study underwent a comprehensive design, evaluation, optimization, and selection process. This involved a parametric Computational Fluid Dynamics (CFD) analysis where various configurations of nozzle length (*L*) and diameter (*d*), as depicted in Figure 1, along with different internal structures, were assessed as depicted in [21]. Factors such as eddy viscosity, air velocity profiles, and streamlines were taken into account, alongside the flow regime. Subsequently, those configurations exhibiting predicted homogeneous behavior were fabricated and subjected to image analysis to evaluate their jet morphology [21]. This step served to validate the CFD results and compare different internal geometry designs. Following this validation, the parametric design of the selected nozzles, comprising four main components, an air inlet body, a central body, an air outlet cap, and an ellipsoidal body serving as the internal core, was evaluated in the present research. The ellipsoidal body was divided into two parts, ellipsoidal body 1 and ellipsoidal body 2, both integrated into the central body of the nozzle as a single unit, as described in Figure 1. To ensure precise alignment of the components forming the central ellipsoidal body, a centering cylinder was incorporated. Assembling the parts was achieved using M3 bolts and M3 lock nuts, securing the bodies along with the outlet cover symmetrically. Additionally, O-rings (020) were utilized as seals to prevent compressed air leakage radially into the cavity. The O-rings were situated in four grooves: the first within the inlet body, the second and third within the central body, and the fourth within the air outlet cover. Figure 1a illustrates a cross-sectional view indicating the expected operating schematic, while Figure 1b displays an isometric view of the assembly with transparency, revealing the integration of the internal elements. An exploded view of the nozzle is shown in Figure 1c, providing insight into the assembly sequence. The design was conceptualized to accommodate dimensional variations in the cavity’s length and the outlet orifice’s diameter. These variations are essential for an experimental design explained in the subsequent section. By adjusting these parameters, we generated three central bodies with different lengths and three outlet caps with varying outlet diameters for the experiments.

This design allowed for nine nozzle configurations resulting from the combination of the three central bodies and the three caps for the outlet body. In Figure 1d, a cross-sectional view illustrates the overall internal dimensions of operational nozzle cavity 1, along with a description of the selected geometric variation parameters for this study. For this case, the parameter *L* = 16.88 mm, resulting in a total length of the central cavity of 33.12 mm, and an outlet diameter *d* = 2 mm. Table 1 provides a description of the nine resulting nozzle configurations, including their dimensional parameters as illustrated in Figure 1d.

### 2.2. Manufacturing of the Parametric Nozzle Parts

#### 2.2.1. First Stage: 3D Printing of the Nozzle Components

The fabrication and assembly of the parts were carried out in two stages. The first stage involved the 3D printing of the main parts, and the second stage focused on the assembly. The 3D-printed parts were manufactured using Onyx material and a 3D Printer Markforged two both from the brand Markforged Inc. (Billerica, MA, USA). Before proceeding with these steps, bilateral geometrical tolerances were determined for the depth and internal diameter of the housing groove in the bodies and the nozzle cap. The slot size was precisely dimensioned to allow the O-ring to deform against the cavity walls when the bolts are tightened. This design choice ensures effective sealing during operation.

**Figure 2:**
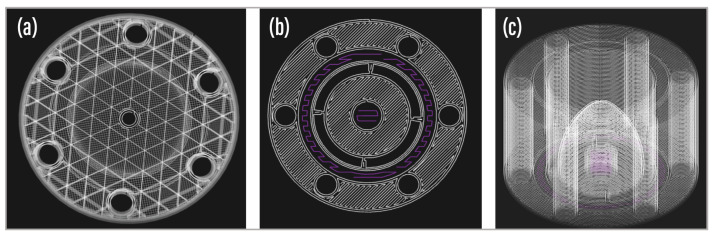
Visualization of print parameter settings in Markforged software. (**a**) Two-dimensional view showcasing the triangular fill pattern on the outlet cap. (**b**) Visualization depicting the layer height pattern at the base of one nozzle body. (**c**) Three-dimensional view presenting the second nozzle body with a hidden fill pattern.

**Figure 3:**
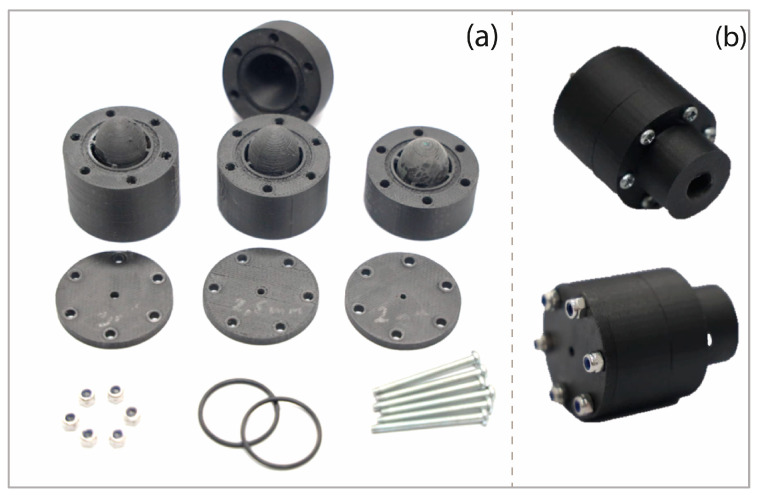
(**a**) Nozzle parts with central bodies assembled to the ellipsoidal bodies. (**b**) NZL3 nozzle prototype assembled.

Consistent design parameters were universally applied to all the parts within the nozzle. Figure 2 illustrates the printing topology visualization from the Markforged two additive manufacturing software. Specifically, Figure 2a presents a preview of the fill pattern on the air outlet cap, while Figure 2b displays the layer height pattern at the base of the center body. Additionally, a 3D view showcases the wall thickness in the center body. Figure 3a shows the fabricated parts of the nozzle along with the other components of the assembly.

#### 2.2.2. Second Stage: Post-Processing of Printing and Assembly

After 3D printing, the parts were carefully freed from supports in the grooves of the central bodies, entry bodies, and sectioned ellipsoidal bodies. Using tweezers, the surfaces were gradually refined with 1000-grit and 1500-grit sandpaper to achieve smooth finishes. Caliper checks confirmed precise dimensions and tolerances. To enable a 1/8 NPT union on the nozzle inlet body, we tapped the air inlet hole with specified dimensions, securing the part in a mechanical press. The assembly occurred in three steps: (1) On a stable surface, we placed the nozzle’s central body with the ellipsoidal body’s exposed hole 2 and applied epoxy adhesive (Loctite super bonder) to the inside of the hole. Gently, we inserted the centering cylinder over the hole using a rubber hammer, and then added adhesive to the exposed surfaces of the cylinder and ellipsoidal body 2, gluing ellipsoidal body 1 similarly. (2) With the inlet body in a vertical position, we positioned the first O-ring in its groove and placed the central body from step 1. The second O-ring was inserted into the front groove, and the air outlet cover was added. (3) Ensuring concentric alignment, we held the assembly vertically and symmetrically secured all nozzle components with screws. This facilitated the assembly of nine nozzle configurations, defining the parametric prototype for experimental evaluation in the next chapter. Figure 3b shows the assembled NZL3 nozzle prototype in two isometric views.

### 2.3. Experimental Evaluation of the Impact Force

An experimental setup to measure the impact force of the pulsed air from each nozzle configuration was developed. Pneumatic and control elements were integrated to develop the pulsed air excitation nozzle device. The device integrates an air compressor (IRVINE OFS750-25), a pneumatic maintenance unit (FESTO MSB6), a 3/2 solenoid valve (TG2321-06-DC24V), and the nozzle to be assessed. Polyurethane 6 mm hoses were used to connect the compressor air outlet to the pneumatic maintenance unit inlet and the pneumatic maintenance unit outlet to the solenoid valve inlet. The hoses were connected by means of push-in fittings.

**Figure 4:**
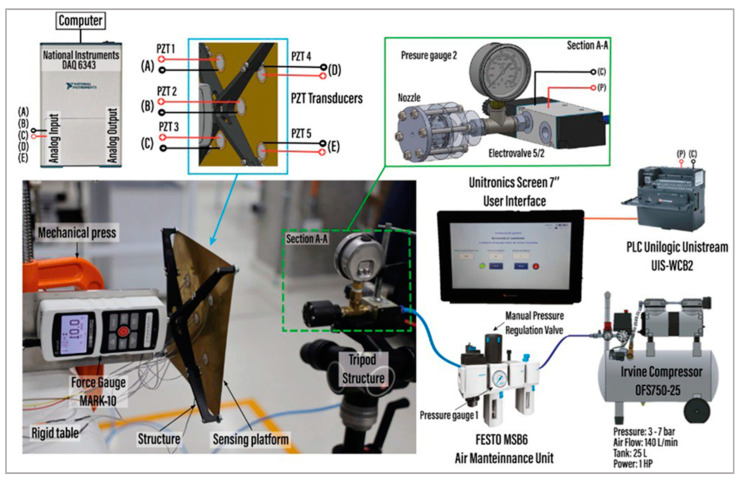
Experimental setup for evaluating the impact force generated by the pneumatic actuator device across the nine nozzle configurations.

The pneumatic actuator nozzle device assembly involved connecting the nozzle to the solenoid valve outlet using two unions with 1/8 NPT external threads and a tee with a 1/8 NPT internal thread, which also accommodated a pressure gauge. The assembly was securely fixed to a support tripod using a C-type press, as shown in Figure 4. A PLC (Unilogic Unistream UIS-WCB2) was implemented, where the coil of the 3/2 solenoid valve was connected to the digital output of the PLC and the negative port. Additionally, a 7-inch display was installed and connected to the PLC UPS. A Ladder language program was developed using the manufacturer’s Unilogic software, creating a user interface to define the opening frequency and the number of air discharges in the solenoid valve. For measuring the impact force level of the device, a system was implemented, comprising a brass platform coupled to a MARK-10 force sensor via a PLA printed structure. The brass platform held five piezoelectric transducers bonded with epoxy adhesive (Loctite super bonder) and positioned as illustrated in Figure 4. The piezoelectric transducers were connected to the analog input of a National Instruments NI 6343 acquisition board to acquire vibration signals. MATLAB R2021 software was used to develop a code for data collection. During the experiment, the nozzle was positioned at a 25 cm separation from the brass platform of the measurement system, with the air outlet hole pointing towards the center of the brass plate. Force and voltage measurements were acquired after conducting shooting tests for each of the nine nozzle configurations. This experimental design aimed to understand the influence of varying cavity length and nozzle outlet diameter on the impact force produced.

**Figure 5:**
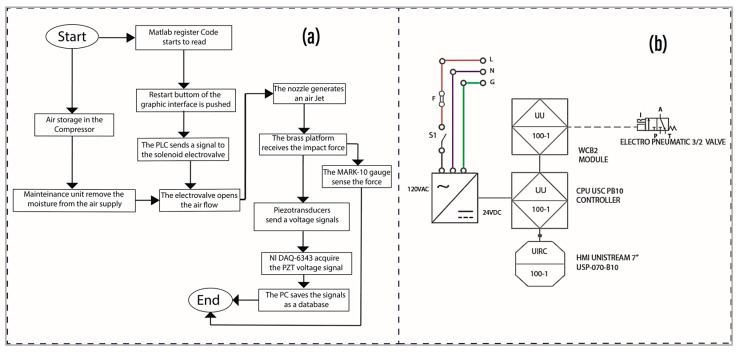
(**a**) Flowchart illustrating the experimental process. (**b**) Schematic diagram of the solenoid electro valve for pulse temporization.

This study evaluated several variables to understand their influence on the performance of the nozzle prototype. These variables included geometric and cavity parameters, such as length and diameter, which were systematically varied and are detailed in Figure 5. By altering these dimensions, this study aimed to investigate how changes in the physical characteristics of the nozzle affected its ability to generate force. Furthermore, this study examined the impact of different pressure levels on force generation. Tests were conducted across a range of pressures, from 5 to 7 bar with 0.5 bar intervals, to assess how variations in pressure influenced the nozzle’s performance. Higher pressures typically result in increased force output, but this study sought to quantify this relationship and identify optimal pressure conditions for maximizing force generation.

Additionally, this study explored the effect of frequency on force generation. Three specific frequencies, 40, 42, and 45 Hz, were selected based on prior vibration analysis of Coffea arabica L. var Castillo coffee beans. These frequencies were chosen since they corresponded to mechanical resonances observed in the beans, suggesting that vibrations induced at these frequencies would be particularly effective in generating force [23,24]. By examining force generation at these frequencies, this study aimed to determine the most effective frequency range for maximizing impact force on the target structure. Overall, by systematically evaluating these variables, this study aimed to understand the factors influencing nozzle performance comprehensively. This would be crucial for optimizing the nozzle prototype’s design and enhancing the contactless excitation technology’s efficacy in various applications, including selective fruit harvesting and others.

The experiment controlled the air outlet pressure through a regulating valve in the pneumatic maintenance unit. This unit not only regulated the working pressure but also removed moisture from the air compressor. The experiment began by executing a MATLAB code to enable the board for data reading, followed by activating the solenoid valve from the user interface to trigger the impact measurement system board. Concurrently, the maximum peak force was recorded on the MARK-10 force sensor display. A sampling rate of 1000 Hz and a data acquisition time of 10 s were configured in the MATLAB code to capture the vibration signal with the piezoelectric transducers. The measurement data, dependent on nozzle, pressure, and firing frequency, were then collected and stored in databases. The flowchart for this experimental process in Stage 1 is described in Figure 5a.

To ensure precise excitation frequencies, a high-precision and reliable pneumatic valve and controller were employed. The schematic diagram designed for trigger control is shown in Figure 5b. The setup involved a 24VDC/1A supply, a Unistream CPU, a Unistream 7” HMI, a Unilogic UIS-WCB2 I/O module (with NPN transistor outputs capable of handling up to 100 kHz switching frequency), and a 3/2 pneumatic solenoid valve (TG2321-06-DC24V). Together, these components were used to control the trigger frequency of the device. A program was developed in Unilogic Unistream software to manage the trigger frequency, number of shots, and test duration. The output frequency was programmed using a TP pulse timer, which sent the switching signal to the solenoid valve for trigger control. It is important to highlight the reliability and robustness of the system and the control program, as the switching times of the transistors are in the order of microseconds (0.4 us for opening and 3.4 us for closing), and the maximum operating frequency is 100 kHz. Therefore, recalibration in terms of frequency is unnecessary, as we can rely on the manufacturer’s specifications, as shown in Figure 5b.

## 3. Results

### 3.1. Experimental Measurements for the Force and Vibration

#### 3.1.1. Force Measurements

**Figure 6:**
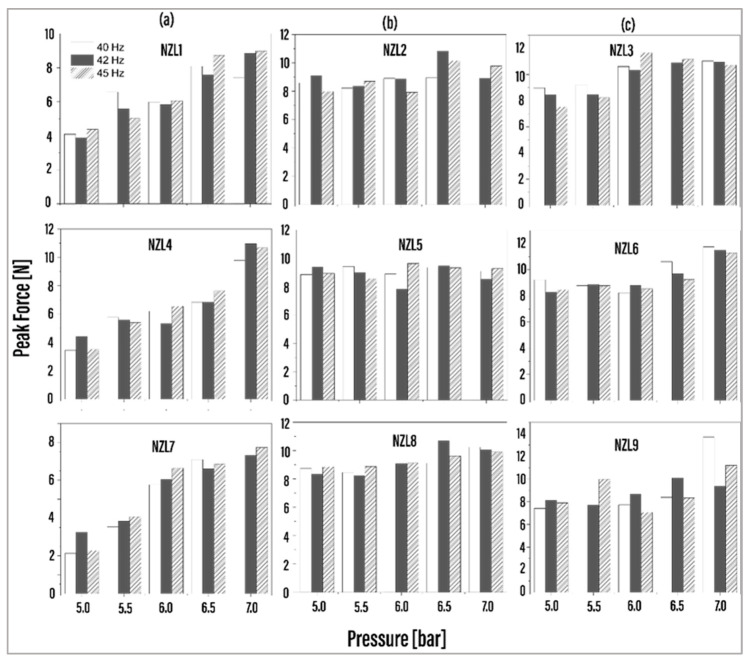
Force measurements obtained with the MARK-10 sensor. (**a**) NZL1, NZL4, and NZL7. (**b**) NZL2, NZL5, and NZL8. (**c**) NZL3, NZL6, and NZL9.

The force measurements obtained for the impact tests performed in the experimental stage are shown in Figure 6. Each graph represents the three firing frequencies (40, 42, and 45 Hz) plotted against the five pressure levels (5–7 bar) for the nine nozzle configurations. Notably, distinct force behavior trends emerged at each pressure level. Nozzles NZL4 and NZL7 exhibited a linear increase in force from 5 to 7 bar, as represented in Figure 6a. In contrast, nozzles NZL1, NZL2, NZL5, and NZL8 demonstrated similar force levels close to 10 N across all frequencies and pressure states. Meanwhile, nozzles NZL6 and NZL9 displayed similar force behaviors between 5 and 6 bar, with a noticeable increase in force observed from 6.5 bar for NZL6 and from 7 bar for NZL7. Notably, NZL3 showcased the highest force levels between 6 and 7 bar, with nearly identical force levels nearing 12 N, while the lowest levels were observed between 5 and 5.5 bar, with values approaching 10 N. Across the studied frequency levels, most nozzles exhibited comparable force measurements at nearly all pressure levels, with a few exceptions. For instance, at 7 bar, NZL9 exhibited a significant increase in force at 40 Hz compared to the measurements at 42 and 45 Hz.

A unifactorial design was employed in our study, with the nozzles as the study factor, and with each level representing the pressure (measured in bar). All experiments were replicated three times to ensure reliability. Peak force served as the response variable for statistical analysis, conducted across three different frequencies. Analysis of variance (ANOVA-one way) was utilized to analyze all data, with significance determined at a probability level of *p* ≤ 0.05. Additionally, post hoc analysis was conducted using the multiple-range Least Significant Difference (LSD) test in R software version 4.3.2 (2023 The R Foundation for Statistical Computing, Vienna, Austria). Notably, statistical analysis revealed significant differences in behavior at 5 (*p*-value = 0.032) and 6 (*p*-value = 0.015) bars of pressure, with the force recorded exhibiting a more uniform pattern. Analyzing the impact capability and force behavior of each configuration, some initial conclusions could be drawn. For NZL4 and NZL7 nozzles (column Figure 6a), increased pressure is required to raise the force. Conversely, nozzles NZL1, NZL2, NZL5, and NZL8 can operate with similar force levels across all frequencies (column Figure 6b). Moreover, NZL3 exhibits high force levels at 5 and 5.5 bar and shows a sensitivity in force increase from 6 bar, while NZL6 and NZL9 demonstrate this sensitivity from 6.5 bar (column Figure 6c). These behaviors highlight the influence of geometrical changes on the measured impact force. However, a detailed numerical analysis of the experimental data is required, which is discussed in the next section.

#### 3.1.2. Voltage Measurements

**Figure 7:**
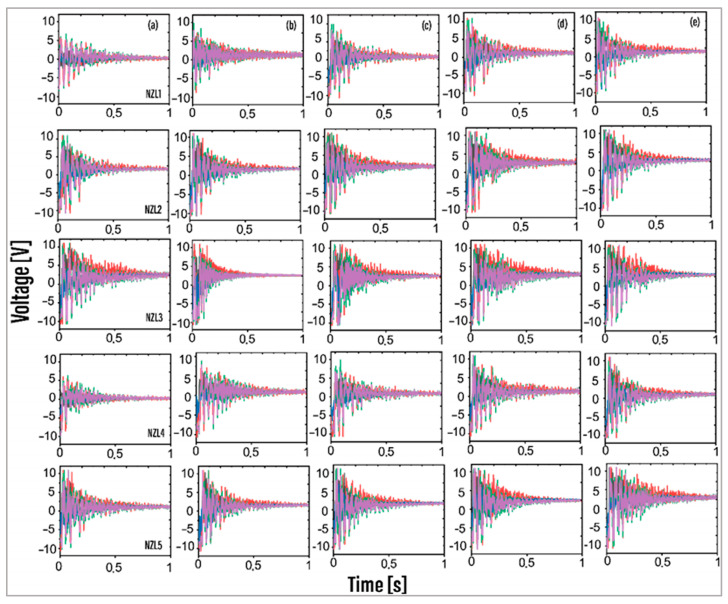
Vibration voltage measurements obtained from NZL1-NZL5 piezoelectric transducers. (**a**) 5 bar. (**b**) 5.5 bar. (**c**) 6.0 bar. (**d**) 6.5 bar. (**e**) 7 bar. Only five nozzles are shown. Black: PZT1; Red: PZT2; Blue: PZT3; Green: PZT4; Purple: PZT 5.

The voltage measurements obtained from the piezoelectric transducers (PZT) are illustrated in Figure 7. For each vibration pulse generated, 2281 data points were collected on the measurement signal to have the same sampling. The forced vibration signal starts with higher amplitude until its attenuation due to energy dissipation.

For nozzles NZL1, NZL2, NZL3, NZL5, NZL6, NZL8, and NZL9, the maximum voltage amplitudes consistently registered at 16 V across all pressure levels. However, for nozzles NZL4 (at all pressures) and NZL7 (from 5 to 6 bar and at 7 bar), the lowest vibration amplitudes were observed. Interpreting the voltage plots requires cautious consideration, as achieving conclusive depth in the analysis is challenging. The dominant signals originate primarily from piezoelectric transducer 5, positioned in the center of the plate, heavily influenced by air impingement from the nozzle. However, some cases indicated that piezoelectric 2 exceeded the vibration amplitude of all signals in certain instances, possibly due to deviations in the jet path.

### 3.2. Assessment of Experimental Data, Optimization, and Nozzle Design Selection

**Figure 8:**
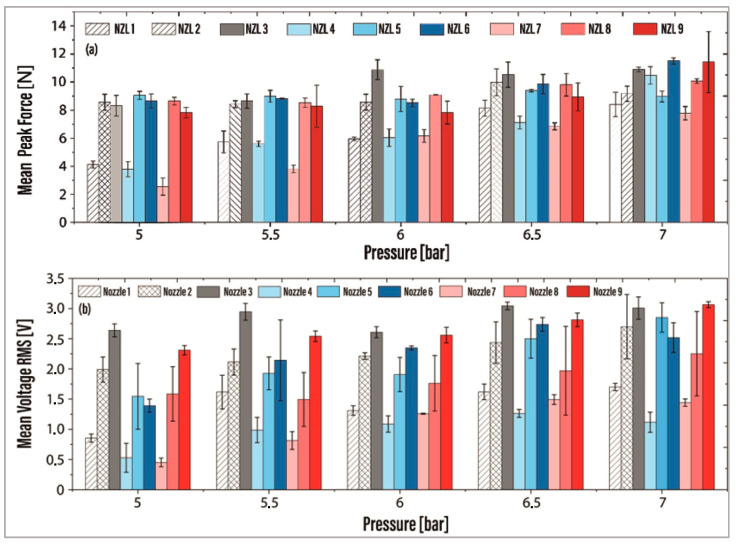
(**a**) Mean peak force measured between 5 and 7 bar of pressure and (**b**) mean RMS voltage measured between 5 and 7 bar for the nine nozzle configurations.

The mean force data (40, 42, and 45 Hz) for the nine nozzles at all pressure levels are shown in Figure 8a. Nozzles NZL2, NZL3, NZL5, NZL6, NZL8, and NZL9 achieved the maximum force levels at 5 and 6.5 bar. Notably, NZL3 outperformed the other configurations in force at 6 and 6.5 bar. At 7 bar, the maximum average force intensity was reflected by NZL3, NZL6, NZL8, and NZL9 nozzles. However, it should be noted that NZL9 exhibited a wide standard deviation due to the force increase at 40 Hz in the experimental measurements, observed at both 5.5 bar and 7 bar. A similar trend was evident for NZL9 at 5.5 bar with a pronounced standard deviation. Analyzing the results from Figure 8a, NZL3, NZL5, and NZL8 exhibited efficient behavior at 6 and 6.5 bar.

The root mean square (RMS) of the voltage signals was determined for each nozzle’s voltage measurement, and the mean value between the RMS values at 40, 42, and 45 Hz was calculated (see Figure 8b). At all pressure levels, nozzles NZL2, NZL3, NZL5, NZL6, NZL8, and NZL9 demonstrated the highest RMS values. Notably, at 5 bar, the largest deviations in the data were observed for NZL4, NZL5, NZL6, and NZL8; at 6 bars for NZL6 and NZL7; and at 7 bars for NZL2 and NZL8. In contrast, designs NZL1, NZL4, and NZL7 displayed the lowest levels in RMS voltage.

It is evident from Figure 8 that NZL3 emerges as a suitable design alternative, exhibiting higher RMS voltage levels at all pressures. This finding suggests that the actuating device could effectively operate at pressures below 7 bar while still generating comparable vibration energy levels. Upon analyzing the results, it becomes evident that NZL2, NZL3, NZL5, NZL6, NZL8, and NZL9 operate with high levels of force and voltage across all pressures. From an energy efficiency standpoint, it is more favorable to operate below 7 bars, as indicated by the force levels exhibited. To further optimize the design and maximize the force as a response variable, a Design of Experiments was conducted using Minitab 19 software. The analysis incorporated two factors (cavity length “*L*” and outlet diameter “*d*”) with two levels (pressure 5 bar and 6 bar) and evaluated their influence on the response variable (force). Two response surface regressions were performed with a confidence level of 95%. Equations (1) and (2) represent the characteristic surface equations approximated to the spatial points for 5 and 6 bar, corresponding to a second-order model. It is also apparent from this plot that NZL3 is a suitable design alternative since it shows higher RMS voltage levels at all pressures. This also suggests that the actuating device could operate at pressures below 7 bar, generating almost equal vibration energy levels.

Analyzing the results, NZL2, NZL3, NZL5, NZL6, NZL8 and NZL9 operate with high levels of force and voltage at all pressures. From an energy point of view, it is more efficient to operate below 7 bars as indicated by the force levels exhibited. An experimental design was performed to evaluate the optimal combination of parameters to maximize force as a response variable using software Minitab 19. The analysis was structured with two factors (cavity length “*L*” and outlet diameter “*d*”) for two levels (Pressure 5 bar and 6 bar) and analyzed their influence on the response variable (Force). Two response surface regressions were performed for a confidence level of 95%. Equations (1) and (2) describe the characteristic surface equations approximated to the spatial points for 5 and 6 bar, which correspond to a second-order model.

F (5 bar) = −77.04 + 0.538*L* + 59.9*d* − 0.0199^−2^ − 11.51*d*^2^ + 0.1060*Ld*, (1)

F (6 bar) = −40.7 + 0.217*L* + 35.2*d* + 0.0119^−2^ − 5.02*d*^2^ + 0.326, (2)

**Figure 9:**
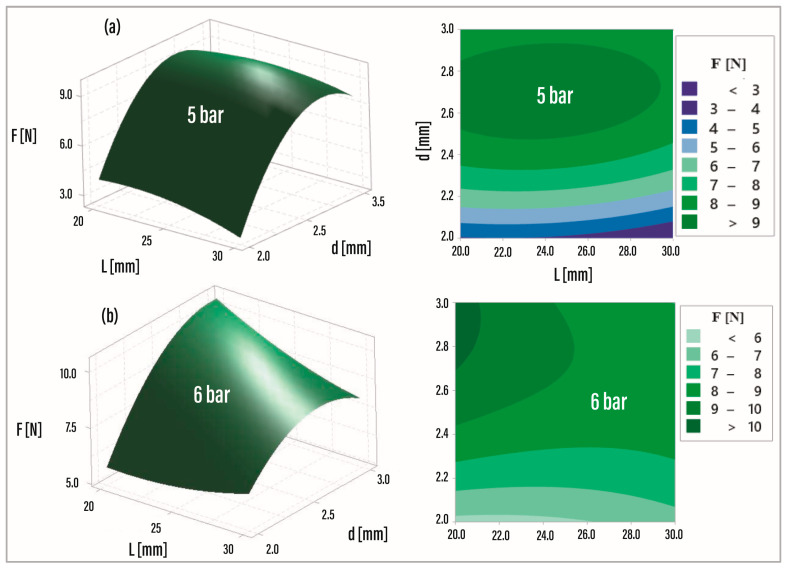
Response surface from the design of experiments. (**a**) Response surface at 5 bar (3D-2D graph). (**b**) Response surface at 6 bar (3D-2D graph).

The response surfaces and contour plots corresponding to Equations (1) and (2) are presented in Figure 9a,b, respectively. At 5 bar (see Figure 9a), an experimental zone is observed with parameter values ranging from 2.5 to 2.9 mm for *d*, and from 16.88 to 25.88 mm for *L*. At 6 bar (see Figure 9b), the zone lies between 2.7 and 3.0 mm for *L*, and between 16.88 and 18.0 mm for *d*. The contour plots indicate that forces above 9 N can be generated at 5 bar, and forces above 10 N at 6 bar, within the regions of interest.

The null hypothesis of this experiment is that the impact force is not influenced by the parameters (*d* and *L*). The 5-bar model showed that factors *d* and *d*^2^ have the greatest influence on the response variable, with values of 0.01 and 0.02, respectively. In contrast, factor *L* exceeded the significance level with a value of 0.121. The level of significance of these factors and their combinations was validated with a statistical significance line of 3.18, considering a significance level α = 0.05.

At 6 bar, factor *d* exhibited the greatest influence on the response variable, with a value of 0.014. Meanwhile, both *d*^2^ and *L* exceeded the significance level, with values of 0.091 and 0.279, respectively. The significance of these factors and their combinations was validated using a statistical significance line of 3.182.

Table 2 shows the regression summary for the response surfaces. At 5 bar, it presented a value of 99.19% and at 6 bar, 92.98%. These values indicate that the data fit the models well, although the 5-bar model presented a higher linearity. According to these results, the models comply with the three assumptions of ANOVA: normality, independence, and homoscedasticity. Finally, in this stage, the optimization values of the response variable were obtained for the most successful configuration of the factors to maximize the force at 5 and 6 bar (see Table 3).

It is important to highlight that this study does not revolve around the force sensing system but the design and evaluation of different proposed nozzles. However, to compare all nine nozzles under the same criteria, we developed a force sensing system. Throughout the experiments, we ensured that the structure remained in the elastic region during gas impacts, avoiding any plastic deformation that could affect the baseline measurement of peak force vs. RMS voltage. The calibration system consistently provided accurate measurements.

A correlation analysis between the mean force and the mean RMS voltage was performed to understand their relationship. We observed only one outlier, and for force values over 8 N, there was a slight data mismatch within the 95% confidence interval. Despite this, our analysis revealed a highly significant positive relationship between force and voltage, as indicated by the low *p*-values. Thus, our force-sensing device is valuable in determining the force magnitude between the different proposed nozzles.

## 4. Discussion

The absence of contactless excitation technologies tailored to stimulate delicate structures like fruits and insects presents an intriguing area for exploration and innovation within agricultural practices. While existing fruit harvesting methods have successfully employed direct-contact low-frequency vibration technologies across a range of crops, including apples, citrus fruits, cherries, and olives [25,26], there remains untapped potential for advancements in this domain. For instance, in the coffee industry, where mechanized and semi-mechanized harvesting methods are gaining traction, particularly in coffee-producing regions like Colombia and Brazil, there are evident challenges with current technologies. Brazil’s adoption of mechanized coffee harvesting aims to enhance operational efficiency [27], while organizations like Colombia’s Coffee Research Center (CENICAFE) have developed portable mechanical devices to assist in coffee harvesting [28]. However, these technologies often fall short, resulting in the unintended detachment of green fruit and leaves during harvesting, thus impacting the quality and selectivity of the process.

In response to these challenges, our study seeks to explore the potential of air jet excitation nozzle devices in facilitating fruit detachment, focusing specifically on crops such as coffee, olives, oranges, and berries. While previous research has primarily focused on characterizing dynamic properties using contactless excitation methods for drying and harvesting [29,30,31], our experiments aim to validate the device’s efficacy in inducing specific vibration modes conducive to fruit detachment. Nevertheless, there are numerous promising paths for future research. For instance, investigating deeper into the fluid mechanics of the air flow expelled from the nozzle could yield valuable insights into optimizing force generation as well as the vibration shapes on the branch [29,32,33,34]. This could involve a comprehensive investigation into the relationship between turbulence and velocity parameters and their impact force capability, thereby enhancing the understanding of the underlying mechanisms driving the nozzle performance. Furthermore, exploring various combinations of geometrical parameters within the nozzle cavity holds potential for developing more refined and optimized models capable of precisely concentrating force on the target at user-defined shooting distances [35,36]. Additionally, examining different profiles of the ellipsoidal core could provide insights into its influence on fluid mechanics phenomena and characterize the nozzle behavior in various conditions, harnessing the potential of innovative contactless excitation technologies and enhancing overall efficiency and productivity in fruit cultivation while minimizing environmental impact and maximizing crop yield.

## 5. Conclusions

This study examined the methodology behind designing and manufacturing a prototype nozzle for contactless excitation purposes. It involved evaluating various combinations of cavity length and outlet diameter to optimize air jet excitation while integrating pneumatic equipment and complementary accessories. The instrumentation and control system facilitated programmable shot parameters, enabling precise control over the device’s operation. The impact force capacity was quantified through experimentation, and numerical analysis provided insights into the nozzle’s performance characteristics. NZL3 emerged as the optimal design among the tested nozzles, exhibiting the highest force levels, particularly at 5 and 6 bar pressures. This selection was based on response surface optimization, highlighting its ability to concentrate force efficiently and conserve energy.

Additionally, we propose several opportunities for further research and innovation. First, exploring enhanced nozzle designs, such as multi-stage or variable geometry nozzles, could further optimize force generation and energy efficiency, potentially unlocking new levels of performance and versatility in air pulse excitation technology. Second, investigating material and manufacturing optimization may improve the nozzle prototypes’ durability, performance, and cost-effectiveness. Lightweight yet durable materials and cost-effective manufacturing methods (such as additive manufacturing or advanced machining) should be explored. Third, consider integrating the nozzle technology with robotics for automated fruit harvesting systems. This integration enhances efficiency and precision, creating autonomous harvesting solutions that improve productivity and reduce labor costs. Fourth, developing sensing and feedback systems would allow for the real-time monitoring and adjustment of nozzle parameters. Sensors measuring air pressure, temperature, and vibration could adjust nozzle settings based on environmental conditions. Fifth, explore applications beyond fruit harvesting, such as material handling, surface cleaning, or medical device manufacturing. Adapting technology to different contexts maximizes its impact and addresses a broader range of industry needs. Sixth, an environmental impact assessment should be conducted to evaluate energy consumption, air quality, and soil health, ensuring environmentally responsible practices. Finally, investigating scaling and commercialization strategies could successfully bring the nozzle technology to market, addressing challenges like mass production, regulatory compliance, and market acceptance.

## Figures and Tables

**Table 1:** Geometrical parameters chosen for evaluating the nine configurations of the operational prototype and the nozzle’s geometry.

Nozzle #	*L* [mm]	*d* [mm]
NZL 1	16.88	2.0
NZL 2	16.88	2.5
NZL 3	16.88	3.0
NZL 4	21.88	2.0
NZL 5	21.88	2.5
NZL 6	21.88	3.0
NZL 7	26.88	2.0
NZL 8	26.88	2.5
NZL 9	26.88	3.0

**Table 2:** Summary of the response surface regression.

Model	R-Square	R-Square (Adjusted)
5 bar	99.19%	97.84%
6 bar	92.98%	81.29%

**Table 3:** Optimization of the factors for the maximization of the response variable (force).

Model	*L*	*d*	F [N]
5 bar	20.82	2.71	9.60
6 bar	16.88	2.96	10.38

## Data Availability

The original contributions presented in the study are included in the article, further inquiries can be directed to the corresponding author.
